# Green Environments for Sustainable Brains: Parameters Shaping Adaptive Neuroplasticity and Lifespan Neurosustainability—A Systematic Review and Future Directions

**DOI:** 10.3390/ijerph22050690

**Published:** 2025-04-26

**Authors:** Mohamed Hesham Khalil

**Affiliations:** Department of Architecture, University of Cambridge, Cambridge CB2 1PX, UK; mhmhk2@cam.ac.uk

**Keywords:** neurosustainability, sustainability and public health, well-being, environmental enrichment, brain volume change, natural environment, forest, urban green space, residential greenness, green architecture and biophilic design

## Abstract

As global urbanisation is rising and public health challenges intensify, this systematic review is conducted at a critical time to explore and explain the associations between the parameters of green environments and nuanced adaptive neuroplasticity in the human brain to advance the development of health-focused sustainable cities and buildings in line with the concept of neurosustainability. This review includes studies involving participants of all ages and genders, with no restrictions on health conditions, exposed to green environments regardless of built environment comparisons. A systematic search of Scopus, PubMed, and Web of Science identified relevant studies published up to November 2024. The risk of bias was assessed using the PEDro scale and ROBINS-I domains, and data were analysed narratively due to heterogeneity. Twenty-three studies were included, conducted across the USA, UK, Germany, Spain, Bulgaria, the Netherlands, Japan, and South Korea. Findings reveal that green environments are associated with positive, region-specific brain changes across the lifespan, surprisingly from before birth to late adulthood. While forests showed more significant effects than blue spaces or urban green spaces, residential greenness emerged as a consistently effective exposure, especially within a 300–500 m buffer around home addresses, provided that sky visibility is present. Notably, no studies have examined green architecture or biophilic interiors, although they are more proximal, are associated with greater exposure time, have antagonistic effects, and may potentially limit sky visibility, highlighting a key gap for future research. Limitations include heterogeneity in exposure definitions, methodologies, and targeted brain regions. Still, this review offers a novel synthesis, providing insight into how greening the built environment may sustain not only the planet but also the plasticity of the brain. This review is registered with INPLASY (INPLASY2024110103) and forms part of a doctoral research project funded by the Cambridge Trust in partnership with the Jameel Education Foundation.

## 1. Introduction

The human brain exhibits neuroplasticity, its continuous capacity to adapt structurally and functionally in response to environmental stimuli across the lifespan. However, these neuroplastic changes can be either positively adaptive or negatively maladaptive, depending on whether the environment is enriched, neutral, or adverse. As public health increasingly intersects with neuroscience, there is growing recognition that greening built environments is a way to enrich the environment. This paper systematically reviews the types and parameters of green environments and their potential associations with adaptive neuroplasticity changes. Part of the efforts by the World Health Organisation (WHO) is to adopt a “health-centric” approach to sustainable cities by providing access to green spaces [1,2]. Sustainability for health has established a new frontier recently, where “neurosustainability” emphasises the sustainability of adaptive neuroplasticity changes in the human brain by enriching the environment, part of which is through greening the environment [3]. There is a growing interest in how green cities and architecture can support public health and well-being [4,5,6,7,8,9], and by synthesising current evidence on how green environments influence total or regional brain changes, this review provides a novel neurobiological perspective to inform urban planning and architectural design for improved public health.

## 2. Materials and Methods

The protocol for this systematic review was registered at INPLASY (INPLASY2024110103). The search was conducted across three databases (Scopus, PubMed, Web of Science), and in accordance with the PRISMA (Preferred Reporting Items for Systematic Reviews and Meta-Analyses) statement and checklist.

The search broadens the scope to change across all potential brain regions but uses niche keywords to broaden the search without limiting it. The search included all articles found and published until November 2024, using the following keywords searched for in titles, abstracts and keywords. The first line included (“natural environment” OR “urban green” OR “green space” OR “greenness” OR “residential greenness” OR “tree cover density” OR biophilia OR “biophilic design” OR “green architecture”) and the second line included (neuroplasticity OR plasticity OR “hippocampal volume” OR “hippocampal plasticity” OR “cortical plasticity” OR “cortical thickness” OR “gray matter” OR “grey matter” OR “white matter” OR amygdala OR “brain volume” OR atrophy) with the relationship between both lines defined as (AND). No limits were made to the subjects, fields, or publication dates.

The Population, Intervention, Comparison, Outcome (PICO) framework was used to capture each key element required for the aim of this systematic review. Population (P) is a human subject with no restrictions on age, gender, or health conditions. Intervention (I) is exposure to natural environments or elements from nature (e.g., trees) since some studies may encompass green environments as part of a broader natural environment. For comparison (C), no comparison is set as a prerequisite for inclusion in this systematic review, but the comparison with built environments is accepted if it is available. Outcome (O) is structural or functional plasticity in the human brain.

The screening criteria considered English journal articles, including trials, cross-sectional and longitudinal studies, while grey literature references were excluded (e.g., books, book chapters, conference papers, notes, retracted papers, and reviews). Duplicated articles across the three databases were removed before data extraction. Downloading articles for full-text reading was based on the title, abstract, and keywords assessment based on the PICO-based inclusion criteria. Articles that were eligible for full-text reading were further examined for their reference lists and citations for the potential inclusion of additional relevant articles, if any. The process of data collection, screening, and selection was carried out independently by the sole author precisely following the clearly defined inclusion and exclusion criteria published in INPLASY.

In line with the PRISMA checklist, the risk of bias assessment was initially established to be conducted in this systematic review using the PEDro scale, which would allow the determination of the quality of randomised control trials and the potential risk of bias. Since most of the studies were found to be non-randomised, this systematic review has also used the Risk of Bias in Non-randomised Studies of Interventions (ROBINS-I) tool for a fair overall assessment of all included studies.

A meta-analysis was not feasible given the limited number of studies and heterogeneity. No sensitivity analysis conducted. Data are synthesised narratively based on the findings from the included studies such as green environment type, significant parameters, associated brain changes, age and gender, and effect measures in any reported format. All studies are considered eligible for the synthesis. No risk of bias due to missing results was conducted. Certainty not assessed due to narrative synthesis. Data are presented in organised tables to allow narrative synthesis.

## 3. Results

**Figure 1:**
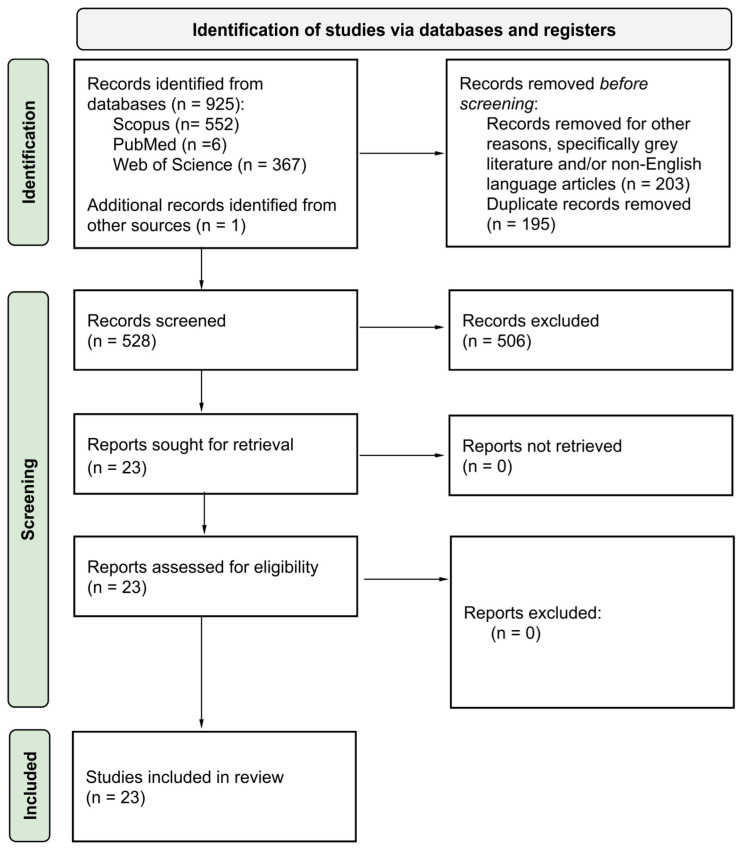
PRISMA flow diagram.

The PRISMA flow chart is shown in Figure 1. Excluded records are studies in the results that do not match the title, abstract and keywords inclusion criteria since they were out of scope but appeared in the search results due to possibly matching only some of the keywords. Included studies (*n* = 23) were diverse in defining a green environment and used different methods to quantify it, and despite this, most studies were focused on older adults, several studies were on adults and young adults, while some were on children, and one focused on the pregnancy phase. This diversity provides a novel perspective on the impact of green environments from before birth until late adulthood but inhibits a meta-analysis.

**Figure 2:**
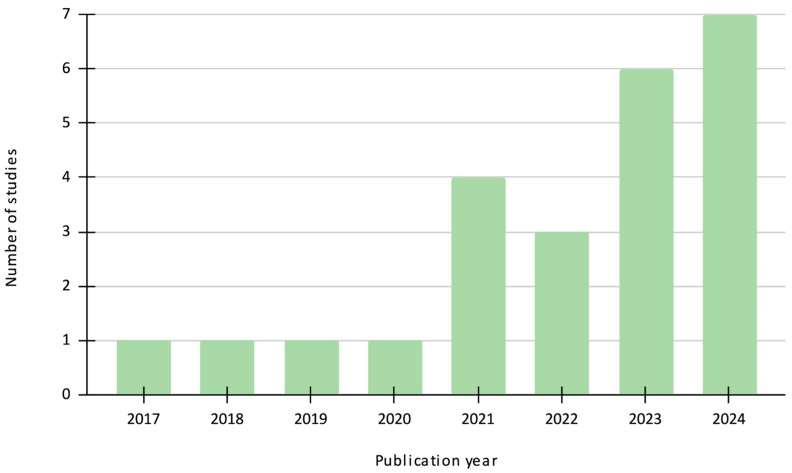
Distribution of included studies per publication year.

**Figure 3:**
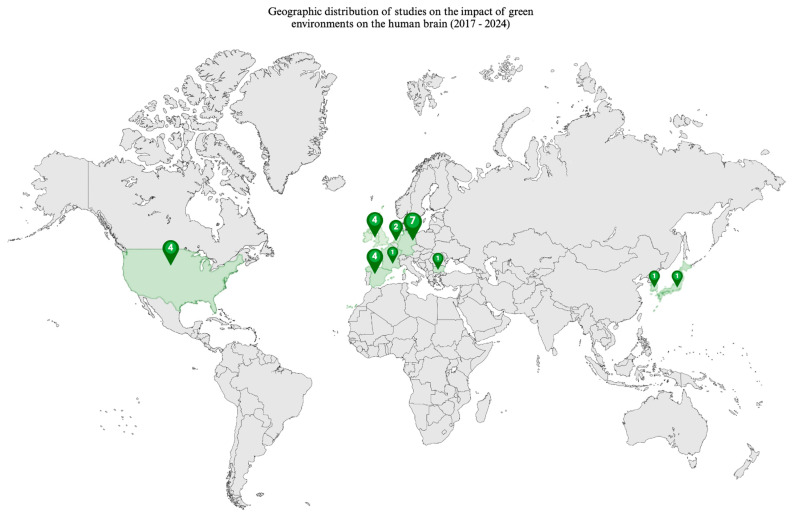
Geographical distribution of the 23 included studies. Some studies used more than one geographic location, while others reported findings of the same study through several publication outputs. The number of studies per country, therefore, is not equal to the number of included studies. The distribution is as follows: Germany (*n* = 7) [10,11,13,21,24,25,26]; Spain (*n* = 4) [16,29,30,32]; UK (*n* = 4) [12,14,15,21]; USA (*n* = 4) [18,19,22,27]; Bulgaria (n = 1) [31]; France (*n* = 1) [17]; Japan (*n* = 1) [23]; Netherlands (*n* = 2) [17,20]; South Korea (*n* = 1) [28].

The included studies spanned publication years from 2017 to 2024, as shown in Figure 2, highlighting a growing recent interest in the impact of the environment on the brain, providing very useful insights for public health. Geographically, as shown in Figure 3, studies predominantly originated from European countries, the United States, the United Kingdom, and East Asia, reflecting a global but somewhat concentrated focus. Table 1 provides a summary of all included studies, while Table 2 presents the risk of bias assessment for randomised trials using the PEDro scale and Table 3 presents the risk of bias assessment for the non-randomised trials using the ROBINS-I tool. All studies show a satisfactory level of risk of bias, except one study with a ‘fair’ score.

**Table 1:** Summary of included studies.

Reference	Sample Size	Age Group	Relevant Aim/s of the Study to this Systematic Review	Results About Brain Changes
Kühn et al. [10]	341	Older adults	Identify geographical characteristics that constitute an enriched environment for the amygdala, pregenual anterior cingulate cortex (pACC), and dorsolateral prefrontal cortex (DLPFC).	Forest coverage (not correlated with wealthier residential areas) was positively associated with amygdala integrity. Urban green and water are not associated with brain structure.
Dettweiler et al. [11]	48	Children	Explore the impact of studying in the forest over one school year on brain development.	Cerebral structural change is best explained by age; however, studying in the forest has a positive direct effect on the maturation of the anterior cingulate cortex.
Xu et al. [12]	156,075; 14,988 (with MRI)	Older adults	Investigate the associations between environments and psychiatric symptoms mediated by brain volume changes.	Found distinct urban environmental profiles (e.g., social deprivation, air pollution, urban density, or conversely protective green spaces) associated with specific psychiatric symptom groups, and observed mediation by regional brain volumes.
Sudimac and Kühn [13]	60	Young adults	Explore the causal relationship between exposure to each the natural and urban environments and the hippocampal formation.	Participants who walked in the forest had an increase in subiculum volume, while no change was observed after the urban walk.
Pu et al. [14]	34,588	Older adults	Examine the associations between air pollution, green space, blue space, and brain volumes, brain age, and frailty.	Green spaces are associated with positive variance in brain volume, gray matter and white matter, particularly in the supplementary motor cortex. Levels of pollution and noise had similar antagonistic effects on brain volumes.
Shang et al. [15]	34,454	Older adults	Examine the associations between greenspace and natural environment exposure on brain volumes.	Positive changes in total brain volume, grey matter, and white matter volumes are influenced by greenness and exposure to nature.
Baena-Extremera et al. [16]	47	Adults	Compare the frontal brain thickness of adults exercising in green outdoor spaces versus urban indoor environments.	The green exercise group exhibited increased thickness in the right anterior cingulate cortex (rACC) and the right superior frontal sulcus (rSFS).
Binter et al. [17]	2820	Prenatal and children	Assess the impact of urban built environment and natural spaces exposure during pregnancy and childhood on white matter microstructure in pre-adolescents.	Greater distance to the nearest major green space during pregnancy was associated with higher whole-brain fractional anisotropy (FA). Higher road-traffic noise exposure mediated the association between lower surrounding greenness and lower FA probably due to releasing stress hormones.
Webb et al. [18]	288 (trauma survivors with MRI)	Adults	Examine how greenspace interacts to affect PTSD outcomes and neural reactivity to reward.	Greenspace exposure was associated with neural reactivity to reward, specifically in the amygdala, and with increased likelihood of being assigned to a resilient PTSD trajectory.
Besser et al. [19]	1260	Older adults	Examine the combined effect of neighborhood greenspace and median income on white matter (WM) grade and ventricle size changes.	The combination of lower neighbourhood income and lower greenspace may be a risk factor for worsening WM grade, but not the greenspace alone dichotomised at median 37% for WM or ventricle grade.
Kühn et al. [20]	95	Children	Investigate the relationship between tree cover density (TCD), open green space (OGS), and sky visibility (sky view factor (SVF)) since birth on brain structure.	Positive association with GM volume in bilateral prefrontal cortex and temporal cortex for OGS; negative association with TCD in frontal and temporal brain regions. SVF was the most significant predictor of GM volume in the medial prefrontal cortex.
Kühn et al. [21]	677	Young adults	Investigate the relationship between tree cover density (TCD) and brain structure.	Current tree cover density at home was positively related to the GM in the right orbitofrontal cortex (rOFC).
Gianaros et al. [22]	699	Adults	Investigate how the features of communities (e.g., green space) relate to brain morphology.	Green space (≥40%) had significant positive indirect effects on cortical and cardiometabolic risks. Indirect effects on hippocampal volume were weaker in effect size.
Tani et al. [23]	476	Older adults	Investigate associations between neighbourhood beauty and regional brain volume.	Subjective neighborhood beauty was positively associated with larger mOFC and insula volumes. The association was stronger in participants living in the mountainous area compared to the downtown area.
Sudimac et al. [24]	63	Young adults	Examine the effect of a one-hour walk in nature (forest) vs. urban environment (busy street) on amygdala activity using fMRI.	Significant decrease in amygdala activity after the nature walk, indicating stress recovery. No significant change after the urban walk.
Sudimac and Kühn [25]	63	Young adults	Examine the effect of a one-hour walk in nature (forest) vs. urban environment (busy street) on amygdala activity.	Found a significant decrease in amygdala activity in women after the nature walk, with no effect in men.
Kühn et al. [26]	207	Older adults	Investigate the association between Urban Fabric (representing urbanicity) and Urban Green (green space) in relation to brain structure (perigenual/subgenual anterior cingulate cortex, p/sACC).	Positive association between Urban Green and p/sACC grey matter volume. Negative association between Urban Fabric and p/sACC grey matter volume. No significant mental health associations were found.
Besser et al. [27]	1125	Older adults	Investigate associations between neighbourhood greenspace and brain-based MRI measures.	No significant association between neighborhood greenspace and hippocampal volume. Borderline association between greater greenspace and lower ventricle grade.
Min et al. [28]	2542	Older adults	Examines the association between residential greenness (EVI) and cortical thickness (global and regional) in cognitively healthy adults.	Significant positive association between residential greenness and cortical thickness in urban populations, particularly in parietal, occipital, and global cortical regions.
Falcón et al. [29]	212	Older adults	Identify structural brain imaging correlates of urban environmental exposures in cognitively unimpaired individuals at increased risk of Alzheimer’s disease (AD).	Exposure to greenness was associated with greater gray matter volume in the middle frontal, precentral, and the temporal pole.
Crous-Bou et al. [30]	228 (with MRI)	Older adults	Evaluate the effect of urban environmental exposures on brain structure of cognitively unimpaired individuals at risk for AD.	Increasing greenness indicators was associated with greater cortical thickness in brain regions known to be affected by AD (entorhinal cortex, temporal, precuneus).
Dzhambov et al. [31]	25 (with MRI)	Adults	Investigate the associations between greenness and neurocognitive functions using brain imaging.	Positively correlated with average cortical thickness across both hemispheres, but a correction method revealed insignificant associations.
Dadvand et al. [32]	253 (with MRI)	Children	Investigate the effect of lifelong exposure to residential greenness on brain structure in children.	Greenness exposure is associated with increased GM volume in the prefrontal cortex and premotor cortex, and WM volume in the cerebellum and prefrontal regions, overlapped with working memory and inattentiveness.

### 3.1. Neurosustainability from the Prenatal to the Ageing Brain Through Green Environments

**Figure 4:**
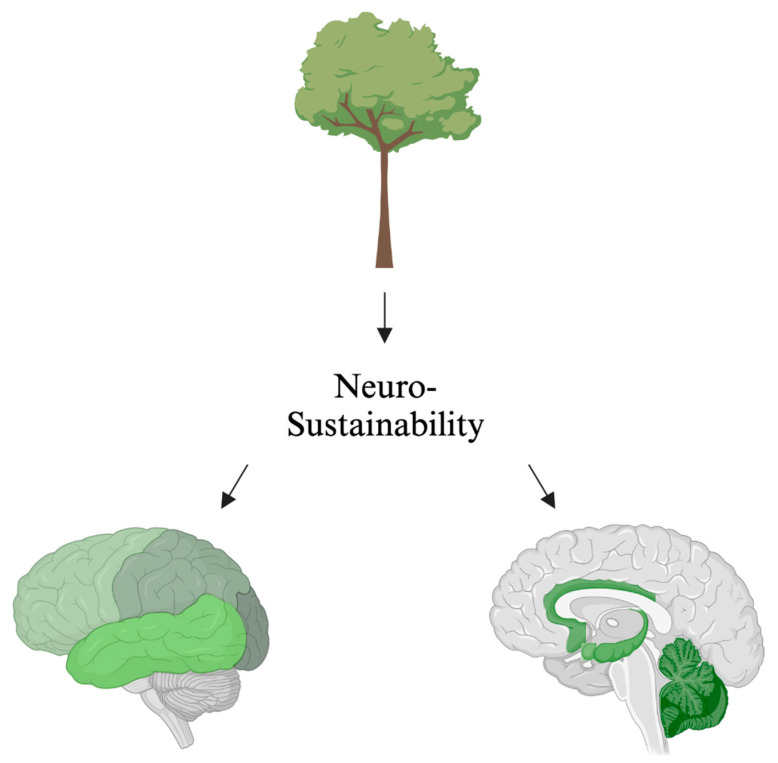
Neurosustainability can be achieved through green environments. The green colours are areas in the brain that this systematic review shows undergo adaptive neuroplasticity responses through exposure to green environments. The brain regions highlighted in green include the amygdala, hippocampus, whole-brain FA, prefrontal cortex, bilateral prefrontal cortex, temporal cortex, parietal cortex, occipital cortex, premotor cortex, precentral cortex, cerebellum, precuneus, ACC, rSFS, rOFC, mOFC, insula, and ventricle grade. This figure does not highlight the age-related or environment-related changes.

First and foremost, the included studies show a positive association between exposure to green environments and adaptive brain changes across various age groups from the prenatal to the ageing brain. Thus, this systematic review can support the theory of neurosustainability [3] (Figure 4). Table 4 shows the distribution of studies and affected brain regions at various stages of life from the prenatal to the ageing brain. Not all studies reported positive outcomes for all brain regions after exposure, which is another limitation in addition to the diverse green environment types, methods and age groups, not facilitating a meta-analysis at this stage.

Exposure to natural environments seems to affect the human brain as early as the prenatal brain during pregnancy. Despite the fact that only one study is available to support this hypothesis, Binter et al. [17] proved through their study, using two populations, that greater distance to public green space during pregnancy was positively associated with higher whole-brain FA at the age of 9 years, suggesting that this is a positive marker of cognitive performance and mental health in adolescence.

Exposure to green environments during childhood is also proven to be effective for adaptive brain responses. On the one hand, Dadvand et al. [32] showed through their study that a higher vegetation index in children’s residential buffer during their upbringing was positively associated with the volumes of their prefrontal cortex, premotor cortex and cerebellum, which were associated with cognitive tests scores. On the other hand, Kühn et al. [20] also studied children’s exposure to greenness but showed that the sky view factor in open green spaces was positively associated with the bilateral prefrontal cortex, temporal cortex, and parietal lobe. In contrast, greenness itself without sky views was negatively associated with the prefrontal cortex, right middle temporal, and left middle occipital gyrus, urging careful consideration of the greenness parameters. In other words, a very high tree cover density in an urban environment will highly inhibit sky views and may result in adverse outcomes.

During adulthood, several studies reported positive associations between exposure to green environments and adaptive brain plasticity responses in total brain, WM and GM volumes [15], precuneus volume [14], rACC and rSFS [16], right orbitofrontal cortex (rOFC) GM [21], subiculum volume [13], and greater amygdala reactivity to reward among PTSD subjects [18]. However, those studies used a variety of definitions and parameters of green environments as well as various exposure durations and focused on different brain region outcomes. This limitation may inhibit immediate generalisability.

Some outcomes were gender-specific. For instance, the reduction in amygdala activity after a walk in the forest was found among women, not men [25], but the reasons are still yet to be understood. Even though those studies may not allow generalisability at this stage, the multifaceted impact of greenness on neuroplasticity and brain health is strongly evident through the limited number of studies.

Last but not least, older adults are shown to benefit dramatically from exposure to green environments. Seven studies confirm this positive relationship [10,23,26,27,28,29,30]. Definitions and parameters varied as well. Also, some of those studies not only focused on older adults through a cross-sectional investigation but also longitudinally, showing some overlaps with studies on adults that need to be further explored.

Synthesising age-related outcomes is not possible at this stage. While promising, several gaps emerge, such as the significant skew of the existing studies towards adults and older adults with less evidence available on children despite the fact that current evidence suggesting that exposure to green environments affects the prenatal brain even before birth, the prevalence of gender-specific brain responses, and varied types and parameters of green environments, which are discussed in the subsequent section.

### 3.2. Green Environment Types, Methods and Associated Adaptive Brain Changes

**Figure 5:**
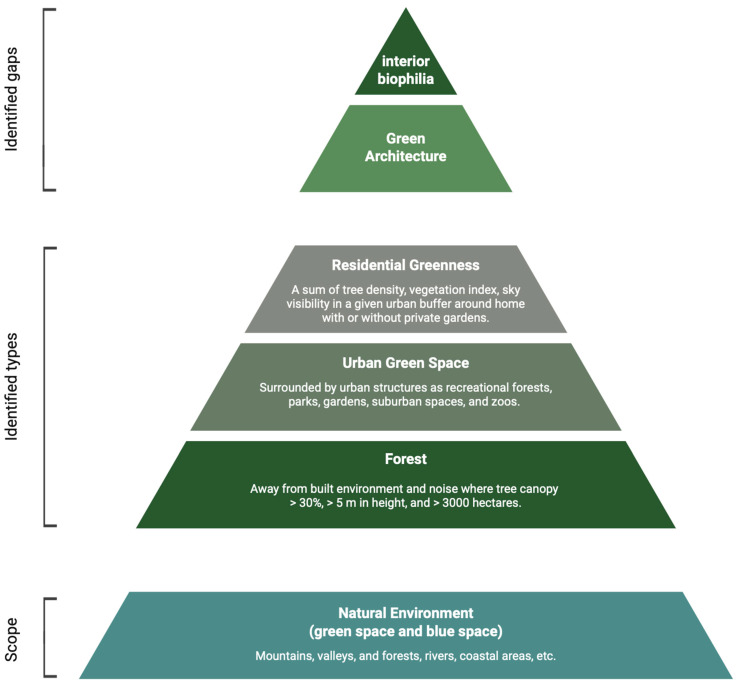
Overview of green environment identification for the impact on brain changes.

Overall, this systematic review supports that exposure to green environments of any type is effective for the adaptive plasticity of the human brain. As illustrated in Figure 5, various types of green environments (forests, urban green space, residential greenness) are identified through this systematic review, but no studies are found on green architecture or biophilic interiors where exposure is more likely to be long-term and is supposed to have more impact on the human brain. The term ‘natural environment’ is also found to be sometimes used as a broader term including not only green spaces but also blue spaces such as rivers and coastal areas.

Table 5 elaborately explains the four key terms that refer to or include a green environment (natural environment, forest, urban green, and residential greenness). However, several methods are used to quantify residential greenness. The National Land Cover Database (NLCD) categorises land and surface features such as developed areas and forests [19,27]. The Normalised Difference Vegetation Index (NDVI) is a measure of greenness derived from land surface reflectance [17,18,23,29,30,31,32]. The Enhanced Vegetation Index (EVI), unlike NDVI, also includes adjustments for canopy background (L), aerosol resistance (C1, C2), and a gain factor (G) [28]. Tree cover density (TCD) data are provided by the European Environment Agency’s Copernicus Land Monitoring Service. It represents the proportion of crown coverage per pixel (20 m × 20 m spatial resolution) and ranges from 0% (areas without trees) to 100% (fully tree-covered areas). TCD measures the vertical projection of tree crowns onto the Earth’s horizontal surface [20,21]. Corine Land Cover (CLC) classifies land into 44 categories, including forests, urban areas, and agricultural land. It uses a minimum mapping unit of 25 hectares for area-based phenomena. It helps capture green areas that may not necessarily include trees [20]. Finally, yet most importantly, the Sky View Factor (SVF) is the amount of sky visible in a given buffer through the tree, building, and sky interactions. Without the SVF, tree density may have adverse outcomes [20].

Buffer radii around residential locations or intervention sites varied from 100 m to 5000 m, with effective measurable impacts commonly identified within narrower buffer ranges (≤500 m). Sky views emerged as a very important parameter through which residential greenness results in adaptive responses, otherwise higher density without sky views results in adverse impacts on the human brain [20].

With the types of green environments being clear, Table 6 highlights the key brain regions evidenced to adaptively respond to the different green environment types, while Table 7 highlights which brain regions change acutely or through long-term exposure to facilitate future research that can address the gaps identified in Table 6.

### 3.3. Green Environment Types’ Effective Parameters for Neurosustainability

The included studies show that the term ‘green environment’ varies significantly as previously explained. Notably, based on the quantifying mechanisms, this term can be classified into a forest, urban green space, and residential greenness, while the broad term ‘natural environment’ may overlap with any of the former three precise terms as well as with non-green spaces such as blue space.

Overall, there seems to be a linear relationship between greenness and adaptive brain changes. Shang et al. [15] provided a significant measure where each 10% increase in greenness at 1000 m was associated with total brain volume, GM, and WM, while each 10% increase in natural environment and other non-green natural features did not explain a significant change in total brain volume. This asserts the insignificance of the broad term ‘natural environment’, and urges an exploration of the effective parameters defining the forest, urban green space, and residential greenness.

Due to the diversity of green environment types, quantification methods, age groups, brain regions affected, and a limited number of studies, it is not possible to identify the effective parameters for neurosustainability through conducting meta-analysis at this stage. Therefore, Table 8 presents all the parameters, significance values, and reported effects from all studies through which a preliminary narrative and semi-quantitative synthesis can be made through this systematic review.

In short, effective parameters for neuroplasticity notably include proximity to dense greenness, the characteristics of natural environments (e.g., forests, urban parks, vegetation index), reduced buffer radius, and minimal exposure to urban stressors such as noise and pollution. This synthesised understanding underscores the potential of targeted greenness interventions as critical components in sustainable urban planning for enhanced neurocognitive health through sustaining the human brain’s neuroplasticity as this process is affected by the environment from before birth and throughout life.

**Figure 6:**
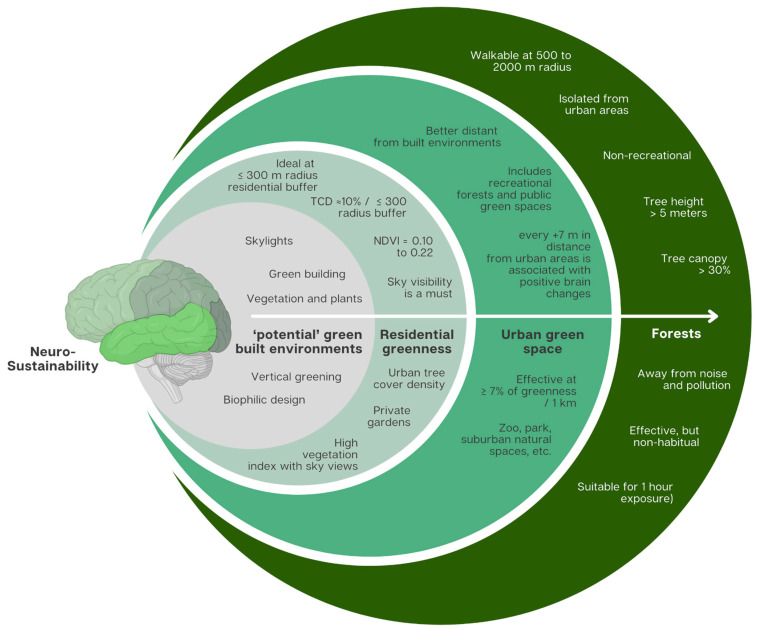
Effective green environment parameters (forest, urban green, residential greenness) for sustainable adaptive neuroplasticity (neurosustainability).

Figure 6 illustrates the synthesis of findings into parameters that distinguish each type of green environment and outlines the minimum parameters proven to sustain adaptive neuroplasticity, though these may be subject to antagonistic variables.

Overall, forests are identified as mainly non-recreational, isolated from urban areas, having tree canopy greater than 30%, and tree height greater than 5 m. Urban green spaces are found to benefit from greater distance from the place of residence, showing that the positive effects of distant forests apply to urban green spaces as well. Given the limited number of studies available, the effective percentage of public urban green space is yet to be generalisable. However, Pu et al. [14] provide percentages that, despite the significance varying dramatically, hint that each 0.5% to 1% increase in green space can explain the variance in brain volume, GM and WM. Residential greenness is based on the radius around the participant’s home address. The data synthesis suggests that studying smaller buffers helps understand the parameters where each 10% increase/1 km was found significant, and both NDVI and TCD methods confirmed those results at 300 m and 500 m buffers, respectively, but found no significance at 1 km, urging cautious reliance on buffers greater than 500 m in general. Residential greenness implies the need for greater sky views as well as through the sky–tree–building relationships for increased sky visibility [20].

#### 3.3.1. Forests

The first study published by Kühn et al. [10] revealed that forests (with ground coverage of tree canopy greater than 30% and tree height greater than five metres), but not urban green, are associated with amygdala integrity. Since then, only four additional studies have been published using the ‘forest’ as a green environment, one tested long-term exposure with low frequency (1 day per week for 1 year), and three of them tested acute exposure. On the one hand, a low frequency of exposure to the forest did not have a noticeable effect among children [11]. On the other hand, Sudimac et al. [24] revealed in their study that, after exposure to stress, a walk in the forest reduced the amygdala activity but it remained stable after a walk in an urban environment, later revealing that this significance is among women, not men [25]. While those three studies enrich our understanding of the interrelationship between the forest and the amygdala plasticity, the fourth study, by Sudimac and Kühn [13], explains the impact of the walk in the forest on the subiculum in the hippocampus, showing an increase in volume after the walk in the forest. Still, several hypotheses are proposed, such as the potential effect of oxygen abundance, the specific impact of variables in the forest (e.g., green colour, sounds, odours, etc.), and environmental novelty. More research is needed to explore the impact of exposure to forests on other age groups, variables explaining the effect of forests, long-term exposure to forests compared to urban environments, how males can benefit from the exposure to forests, and how forests inform built environments.

#### 3.3.2. Urban Green Space

The term ‘urban green’ simply refers to a green environment that is separate from the city yet, unlike a forest, is recreational and is inside the city. Urban green spaces are still representing distinct land uses, and they do not include trees scattered in the city.

Kühn et al. [26] explored the effectiveness of urban green (6.79% at 1000 m buffer), including recreational forests in urban settings, and showed its effectiveness with those given parameters. The authors mentioned that their definition of urban green included land primarily used for recreational purposes, such as parks and gardens, and suburban natural spaces functioning as parks. Forests and green fields are also classified as urban green if they show signs of recreational use and are surrounded by urban structures. For instance, tree-covered parks within cities are considered urban green areas. Therefore, the effectiveness of urban green here may be due to one or more confounding variables. The other study using the term urban green was by Gianaros et al. [22], who showed indirect results and defined green space as a reverse of built surfaces, which prevents synthesising it in line with the previous definition of urban green that itself was accurate but included multiple dimensions of greenness. However, other results can be synthesised. For instance, Pu et al. [14] showed that green space (45.21% ± 21.58% at 1000 m buffer) explained 39.4% of the variance in brain volume, 49% in white matter volume, and 21.7% in grey matter volume, but it was significant to explain the variance only in one brain region. They highlighted the adverse effects of pollution and noise. Binter et al. [17] highlighted the countereffect of noise and found that the greater distance to the public green space was associated with whole brain FA, while noise explained the low greenness and low FA association. Interestingly, a key feature of the ‘forest’ in the series of studies on forests highlighted it was away from noise [13,24,25], suggesting that, beyond the lack of understanding of the effective parameters of forests reducing amygdala activity and increasing subiculum volume, the lack of noise facilitates the working of greenness at the first place.

Urban green space may not be equally effective as forests since one study shows that the amygdala integrity is associated with forests but not urban green [10], but since the antagonistic effects of noise and pollution may explain the difference, besides the difference in the definition of ‘green environment’ itself, urban green spaces are more challenging to test. The surprising positive association between greater distance to public green space and brain changes [17], for instance, can be due to those dynamics.

#### 3.3.3. Residential Greenness

The contrast between distant forests and everyday built environments allows for considering vegetation index measures to blur the boundaries between distant forests and everyday built environments, leading to the emergence of what is known as ‘residential greenness’. This concept represents the tree cover density in a residential buffer. Several studies adopted this approach using different methods and various residential buffers, while Xu et al. [12] included private gardens within this scope.

The normalised difference vegetation index (NDVI) has been used mostly by earlier studies [29,30,31,32] but it is still being used as shown in a couple of recent studies by Xu et al. [12], Binter et al. [17] and Webb et al. [18]. Using the NDVI, positive associations were found specifically at buffer ≤ 300 m (NDVI mean = 0.10 to 0.22) but not at 500 m (NDVI mean = 0.40) residential buffer, highlighting that the density per narrowed buffer area can be more suitable for future research interested in the impact of greenness in urban areas on neuroplasticity and brain health.

Other studies used diverse measures and buffers, but their findings collectively support that a greater buffer area leads to non-significant results. Tani et al. [23] found significant associations in specific brain regions regarding the subjective measure of beauty but not the objective measure using NDVI at 1000 m, unlike other studies. Additionally, Besser et al. [27] used the NLCD at 1000 m and 5000 m buffers but found no significant relationships. The same applies to the study by Besser et al. [19], who worked on a 1000 m buffer and found no significant neuroplasticity outcomes.

The rest of the studies used other methods that worked for buffers greater than 300 m but still not greater than 500 m, which shows the suitability of different methods for more significant areas, yet the focus on a small area buffer in future research remains more promising. Min et al. [28] used the EVI method at a residential buffer of 750 m, finding that some brain regions had significant neuroplasticity associations while others did not. Kühn et al. [21] used TCD, finding significant associations with positive brain changes at 500 m (10.12% TCD) but not at 1000 m (10.79% TCD) or 2000 m (12.02% TCD). Kühn et al. [20] also found significant associations at 200 m (9.41% ± 5.43% TCD) with smaller effects at 500 m (9.34% TCD) but no significant effects at 1000 m (9.25% TCD). Similarly to NDVI, the density per narrowed buffer area, here estimated at ≤ 500 m (TCD ≈ 10%), can be more suitable for future research. Either way, building on the given effective estimates for either the NDVI or TCD within the buffer limits and ranges proven effective is recommended.

## 4. Discussion

This systematic review shows that the human brain’s neuroplasticity positively changes in response to green environments as early as during pregnancy and until late adulthood, which emphasises that greening built environments is important for lifelong neurosustainability [3], and supports the efforts to enhance public health and well-being through the WHO’s plans to develop sustainable green cities for health [1,2] and the United Nations Sustainable Development Goals to plant and protect trees for sustainability and health [33,34,35]. This review shows that the associations between green environments and brain changes are nuanced, depending not only on the type and parameter of the green environment but also on the sensitivity of the brain region and how it functions. While forests are shown to have significant associations with positive adaptive neuroplasticity compared to blue spaces or urban green spaces for the amygdala (associated with emotional regulation), residential greenness shows that tree cover density with sky visibility is key for the cerebrum, but no conclusions can be made on the hippocampus due to its formation complexity as shown to how it changes in response to walking in a very complex way [36], while very little is evidence is found on the changes of the cerebellum, ventricle grade, and whole-brain FA although they benefit from exposure to green environments as well. There is a noticeable gap in the literature where no studies have explored the impact of greening the built environment itself (architectural or interior), knowing that it has antagonistic effects from the design of the built environment itself to the lack of proximal greenery and the maximised exposure in such green-deprived environments.

From a public health perspective, this systematic review suggests that the enhancement of each of the brain regions shown to positively be improved in response to green environments is multifaceted and can reduce population-level burdens of anxiety, chronic stress-related disorders and cognitive decline. However, brain regions do not often function separately, and complex mental health disorders such as borderline personality disorder (BPD) may emerge if multiple brain regions are impaired in response to urban living [37].

At this stage, it is evident that greening the built environment is critically important, and this review suggests that it can be conceptualised across three primary spatial scales, each with a distinct potential to support adaptive neuroplasticity. At the broadest level, the presence of forests as opposed to urban green spaces has been shown to elicit more significant neural benefits, likely due to greater ecological richness, sensory immersion, and biodiversity, but more importantly their isolation from urban environments, which may be justified by the findings that distant urban green space is more effective than proximal ones. At the neighbourhood scale, increasing tree cover density within a defined residential buffer (typically 300–500 m) is critical, particularly when balanced with unobstructed sky visibility, which if absent, shows that tree cover density can have adverse effects. At the most proximate level, and through the insights obtained, integrating green design parameters into architecture and interior environments may have the most significant impact on neuroplasticity given the increased duration of exposure and highest proximity of contact and may even hold antagonistic effects with counteractive forces to green environments. This systematic review enriches the efforts on sustainability, green architecture and biophilic interiors [38,39,40], which can benefit from the nuanced parameters identified through this systematic review. Still, without reducing the adverse effects of urbanism- and built environment-related causes of pollution and noise, the efforts of greening the environment may be of no significant effect since those antagonistic effects work in the opposite direction to the green environment on the same brain region [14,17]. For instance, grey space, in contrast with green space, is shown to adversely impact the amygdala’s functional plasticity [41]. These multi-scalar strategies underscore the importance of embedding greenness throughout the built environment to optimise its neurobiological and public health impact.

Through the parameters obtained through this systematic review, the built environment needs to become green so that it becomes a continuous promoter of neurosustainability for lifelong health and well-being. This systematic review suggests that it can be achieved in different ways through which the built environment can be designed to be effectively ‘green’:

1. Turning buildings into ‘forest’-like tree canopies through but not limited to adding natural vegetation to the building facades and using natural materials such as wood while maximising sky views in urban environments through setbacks. Green façades are shown to contribute to stress recovery and well-being in high-density cities [42], which supports that green building exteriors may have the same restorative effects that forests have on the amygdala and hippocampal subiculum [13,24,25]. Vertical greening is another trend that can be challenged if it is able to promote adaptive neuroplasticity since health and well-being outcomes are not objectively measured or quantified for studies published until October 2022 on vertical greening [43].
2. Maximising tree cover density with sky visibility in urban environments, particularly within 300 radii around home addresses, including private gardens. This conclusion is supported by the findings obtained from this systematic review. The minimum 300 m radius is in line with WHO’s specification that accessibility to a green space should be no more than 300 m linear distance (5 min walk) from home, and with the European Common Indicator of local public open areas given the same 300 m distance from a public open area [44]. However, it appears through this systematic review that residential greenness may perform better than urban green space, which urges urban planners to rethink the parameters needed for a green environment to be effective for neurosustainability.
3. Increasing indoor plants and providing sky views through skylights may be another way to maximise the potential impact of greenness due to the direct contact with plants and the sky since people spend more time indoors. To support this hypothesis, studies show that homebodies have poorer cognitive performance and poorer mental health compared to others who venture into the city [45,46], while greater time spent outdoors is found to be positively associated with grey matter volume in the prefrontal cortex [47], which is in line with the findings of this systematic review.
4. Increasing vegetation in balconies to maximise short- and long-term exposure to green environments. It is not only plants in balconies that allow direct contact with a green environment, but sky visibility is highly present through this architectural liminality. This is a possible hypothesis that may hold significant potential for neurosustainability if it is difficult to test the indoor environment given the architectural design limitations and confounding variables available.

While this review focused specifically on adaptive neuroplasticity, it is important to recognise that green environments exert multi-systemic effects that may operate synergistically to support overall health. Exposure to green environments has been linked to improved cardiovascular outcomes [48], immune function regulation [49,50], enhanced sleep quality [51,52], and reductions in inflammatory markers [53]. These physiological effects may, in turn, interact with neural mechanisms, reinforcing cognitive and emotional resilience through systemic pathways. Future interdisciplinary studies should therefore investigate how these cross-systemic responses co-occur and whether their integration contributes to long-term neurobiological and behavioural health trajectories. Incorporating this broader perspective can support more holistic strategies for urban planning and architectural design aimed at promoting sustainable health across domains.

While the cognitive and neurobiological benefits of green environments are well supported, it is important to consider potential drawbacks or unintended effects. For instance, excessive tree density without sufficient sky visibility has been associated with adverse developmental outcomes in children [20], suggesting the need for balanced spatial design. Moreover, poorly maintained green areas may contribute to allergen exposure [54], or reduced perceptions of safety [55], potentially counteracting restorative effects. Socioeconomic concerns, such as green gentrification [56,57], also raise questions about equitable access to neuroprotective environments. These issues highlight the necessity of context-sensitive, inclusive, and evidence-informed planning that maximises benefits while mitigating risks. Future research should explore how environmental, sensory, and social factors interact to shape both positive and negative brain outcomes in response to greenness.

Last but not least, this systematic review culminates in a non-exhaustive list of potential future research that can build on the identified gaps through this systematic review and provide additional insights into the critical associations between green environments and adaptive neuroplasticity changes:

1. Replicating current studies is important to facilitate conducting a meta-analysis for a specific green environment type, exposure duration, age groups, methods, or brain region-related adaptive changes.
2. Exploring the feasibility of integrating green environments into unexplored geographic contexts is highly needed to open the door for generalisability.
3. Exploring the application of greenness in contexts where forests are not available or in arid environments is challenging and needs more tailored methods.
4. Exploring how to achieve the sky–tree–building balance to not have adverse effects on the human brain as a result of increased tree cover density without sky visibility is the future of urban planning- and design-related research.
5. Exploring the effects of skylights in indoor spaces with or without natural greenery or biophilia on neuroplasticity changes is highly important since people spend most of their time indoors.
6. Exploring the impact of green architecture or biophilic design, architectural or interior, on brain changes after short- and long-term exposure or both can deepen the understanding of the reported dynamics in this systematic review.
7. Exploring the architectural materials that can mitigate the antagonistic effects of noise and pollution that may inhibit the effect of green environments is important.
8. Exploring how urban environments can minimise the effect of grey space or eliminate it is another problem to solve.
9. Exploring the impact of green environments on mental health through neuroplasticity such as depression, PTSD, BPD, bipolar disorder, and others to test whether nature can act as an environmental health-based protocol will further advance our understanding of the impact of green environments on public health.
10. Exploring the underlying molecular mechanisms of how green environments lead to brain volume changes will help explore more creative solutions.
11. Exploring the impact of private gardens as part of residential greenness needs more investigation since they hold greater potential due to their greater proximity within the residential buffer.
12. Exploring the different effects of natural versus artificial or non-natural trees (e.g., liquid trees) that may not capture the profound effects of trees on the human brain is very important at a time when the artificial replication of nature is trending.

## 5. Conclusions

This systematic review provides the first comprehensive synthesis of how exposure to green environments is associated with adaptive neuroplasticity across different stages of human life, from prenatal development through childhood, adulthood, and into late adulthood, emphasising that green environments can achieve neurosustainability. Findings demonstrate that exposure to green environments is associated with beneficial changes in various brain regions implicated in emotional regulation, cognitive control, memory, and sensorimotor function. Three primary green environment types emerged: forests, which consistently showed the most significant neuroplastic effects, particularly for stress-related brain regions like the amygdala and subiculum; urban green spaces, which showed mixed or less consistent effects, often influenced by proximity, exposure quality, or the presence of urban stressors; and residential greenness, which was repeatedly found effective, especially within 300–500 m buffers, and more so when accompanied by sky visibility. Notably, dense tree cover without sky views was shown to have adverse effects in children, underscoring the importance of parameterisation beyond mere vegetation presence. Importantly, no included studies examined the neuroplastic impact of green architecture or biophilic interiors, despite their greater exposure potential and proximity. This represents a critical research gap, particularly given that the majority of human experience occurs indoors. This review affirms that the human brain responds to green environments across life stages in ways that are measurable, region-specific, and dependent on environmental parameters. Urban and architectural design practices should integrate these findings to promote lifelong brain health. Greening the built environment is not only an ecological or aesthetic imperative but also a critical pathway to achieving neurosustainability and sustainable public health through the lens of environmental neuroscience.

## Figures and Tables

**Table 2:** Risk of bias scores for randomised control studies using the PEDro scale.

Study	PEDro Scale Items *										
	1	2	3	4	5	6	7	8	9	10	Total Score
Dettweiler et al. [11]	N	N	N	N	N	N	Y	Y	Y	Y	4
Sudimac and Kühn [13]	Y	N	Y	N	N	N	Y	Y	Y	Y	6
Sudimac et al. [24]	Y	N	Y	N	N	N	Y	Y	Y	Y	6
Sudimac and Kühn [25]	Y	N	Y	N	N	N	Y	Y	Y	Y	6

* PEDro scale Items: 1 = Random allocation, 2 = Concealed allocation. 3 = Groups similar at baseline, 4 = Participant blinding, 5 = Therapist blinding, 6 = Assessment blinding, 7 = <15% dropout rate, 8 = Intention-to-treat analysis, 9 = Between-group differences reported, 10 = Point estimate and variability reported. Scores 0–3 are ‘poor’, 4–5 ‘fair’, 6–8 ‘good’, and 9–10 ‘excellent’.

**Table 3:** Risk of bias scores for other non-randomised studies using the ROBINS-I tool.

Study	ROBINS-I Tool *							
	D1	D2	D3	D4	D5	D6	D7	Overall
Kühn et al. [10]	+	+	+	+	+	+	+	+
Xu et al. [12]	?	+	+	+	+	+	+	+
Pu et al. [14]	?	+	+	+	+	+	+	+
Shang et al. [15]	+	+	+	+	+	+	+	+
Baena-Extremera et al. [16]	?	+	+	?	+	+	+	?
Binter et al. [17]	+	?	+	+	+	+	?	?
Webb et al. [18]	+	+	+	+	+	+	+	+
Besser et al. [19]	+	+	+	+	+	+	+	+
Kühn et al. [20]	+	+	+	+	+	+	?	+
Kühn et al. [21]	+	+	+	+	+	+	?	+
Gianaros et al. [22]	+	+	+	+	+	+	+	+
Tani et al. [23]	+	+	+	+	+	+	+	+
Kühn et al. [26]	?	+	+	+	+	+	+	+
Besser et al. [27]	+	+	+	+	+	+	+	+
Min et al. [28]	+	+	+	+	+	+	+	+
Falcón et al. [29]	+	+	+	+	+	?	?	?
Crous-Bou et al. [30]	+	+	+	+	+	+	+	+
Dzhambov et al. [31]	+	?	+	+	+	+	+	+
Dadvand et al. [32]	+	+	+	+	+	+	+	+

* Domains: D1 = Bias due to confounding, D2 = Bias due to selection of participants. D3 = Bias in classification of interventions, D4 = Bias due to deviations from intended interventions, D5 = Bias due to missing data, D6 = Bias in measurement of outcomes, D7 = Bias in selection of the reported result. Judgement: + = low risk of bias, ? = moderate risk of bias, x = serious risk of bias, ! = critical risk of bias.

**Table 4:** Green environments and its potential promise for lifelong neurosustainability.

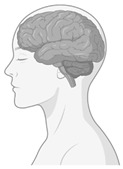 Adults (females)	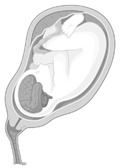 Prenatal	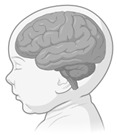 Children	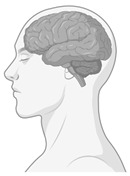 Adults	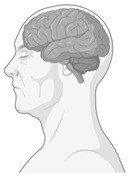 Older adults
Reduced amygdala activity to stress [25].	Higher whole-brain FA [17].	Bilateral prefrontal cortex, temporal cortex, parietal lobe [20]. Prefrontal cortex, premotor cortex, and cerebellum GM, and WM volumes [32].	Bilateral subiculum [13], precuneus [14], total brain, WM and GM [15], rACC and rSFS [16], greater amygdala reactivity to reward [18], and rOFC GM [21].	Amygdala [10,12], cerebellum [12], mOFC and insula [23], p/sACC [26], ventricle grade [27], global cortical, parietal and occipital cortex thickness [28], middle frontal cortex, left precentral cortex, right temporal cortex [29], frontal cortex [12], and cortical thickness [30].

**Table 5:** Definitions of a green environment.

Green Environment	Description
Natural environment	A very broad term defined as including mountains, valleys, and forests excluding urban greenspaces such as city parks [15], or defined as to broadly include water, arable land, and coastal areas, as derived from the Land Cover Map 2007 [16].
Forest	A precise term defining forests as areas with the ground coverage of tree canopy greater than 30% and tree height greater than 5 m, covering over 3000 hectares, without built structures and free from traffic noise [10,13,24,25].
Urban green	A term used to refer to land primarily used for recreational purposes, such as parks, gardens, and suburban natural spaces functioning as parks or zoos where forests and green fields are also classified as urban green if they show signs of recreational use and are surrounded by urban structures [10,26]. For instance, tree-covered parks within cities are considered urban green areas.
Residential greenness/greenspace	A single concept with multiple identification terms, quantifying the percentage of greenness around a residential area in a given buffer. It is defined as the (a) percentage of land categorised as green within specified buffers around participants’ residential addresses, based on the Generalised Land Use Database (GLUD) [14], (b) percentage of ‘impenetrable surface areas’ (roads, rooftops, parking lots) within a census tract, reversed in coding so higher values represent more green spaces [22], or (c) percentage of greenspace coverage within circular buffers around each home location polygon, calculated as an area-weighted average for each participant [15]. Residential greenness may include private gardens [12].

**Table 6:** Green environment type associations with significant adaptive neuroplasticity changes.

Adaptive Neuroplasticity Across Primary Brain Regions	Green environment type					
	Natural env.	Forest	Urban Green Space	Residential Greenness	Green Architecture	Biophilic Interiors
Amygdala	Not sig. for structural integrity [10]	Effective for structural integrity and reduced reactivity [10,24,25]	Not sig. for structural integrity [10]	Effective for greater reward reactivity [18]; and for structural protection [12]	—*	—
Hippocampus	—	Effective for subiculum volume increases [13]	—	Effective for the para-hippocampal gyrus [20]; indirectly effective for total volume [22]; Not sig. [27]	—	—
Cerebrum (frontal, orbitofrontal; prefrontal, parietal, occipital, premotor, precentral, insular or anterior cingulate cortex).	Not sig. for structural integrity or volume changes [10,14]; but effective for volume increase with exercise [16]	Not sig. for structural integrity [10]	Not sig. for structural integrity [10]	Effective for structural protection [12]; and volume increases [14,21,28,29,32]; through sky visibility [20]	—	—
Cerebellum	—	—	—	Effective for structural protection [12]; and volume increase [32]	—	—
Ventricle grade	—	—	—	Effective [19,27]	—	—
Whole-brain FA	—	—	Effective when more distant [17]	—	—	—

* No studies available.

**Table 7:** Exposure to green environments and regional adaptive neuroplasticity.

Adaptive Neuroplasticity Across Brain Subregions	Exposure to a Green Environment	
	Acute	Long-Term
Amygdala	Sig. [24,25]	Sig. [10,12,18]; developmental influence [11]
Hippocampus *	Sig. [13] ‘subiculum’	Sig. [20,22] for total and parahippocampal gyrus; Not Sig. [27]; developmental influence [11]
Frontal/prefrontal cortex	— **	Sig. [12,20,32]
Temporal cortex	—	Sig. [20,29]
Parietal cortex	—	Sig. [20,28]
Occipital cortex	—	Sig. [20,28]
Premotor cortex	—	Sig. [32]
Precentral cortex	—	Sig. [29]
Precuneus	—	Sig. [14]
ACC	—	Sig. [16,26]; age-frequency influenced [11]
rSFS	—	Sig. [16]
OFC	—	Sig. [21]; but for subjective beauty [23]
Insula	—	Sig. for subjective beauty [23]
Cerebellum	—	Sig. [12,32]
Ventricle grade	—	Sig. [19]; borderline sig. [27]
Whole-brain FA	—	Sig. [17]

* Limited or inconsistent results on the hippocampus or different hippocampal subregions urges a cautious interpretation of findings. ** No studies available.

**Table 8:** Parameters, significance, and effects of green environments on brain changes.

Reference	Age in Years: Mean ± SD	Greenness Buffer	Duration	Greenness Definition	Effectiveness Measurements	Brain Region with Sig. Change	Sig.	Coefficients/Effects Associated Neuroplasticity Outcomes
Kühn et al. [10]	70.1 ± 3.89	500, 1000 and 2000 m	≈8 years	Forest, urban green, water	Forest	a. Amygdala b. pACC c. DLPFC	a. 0.010 b. Not sig. c. Not sig.	a. β = 0.232.
Dettweiler et al. [11]	Fifth and sixth graders (≈10–12 years)	—*	1 year (1 day/week)	Forest	Through the given frequency, studying in the forest only had a modest effect on the ACC but was influenced by outliers.	Hippocampus, amygdala, ACC.	—	—
Xu et al. [12]	59.1 ± 8.09; but individuals with neuroimaging are 57.77 ± 7.49	300 m	—	a. Natural environment % vs. built b. Green space % c. Domestic garden % Method used (NDVI)	Protective factors like greenness and destination accessibility are inversely correlated with anxiety (r = 0.10, Pperm < 0.001), mediated by emotion-related brain regions, explaining 1.65% of the variance.	Amygdala, cerebellum and regions in the frontal cortex.	<0.001	—
Sudimac and Kühn [13]	Urban: 28.73 ± 7.49; Nature: 25.90 ± 5.67	—	1 h	Forest	Walk in the forest not urban environment	Bilateral subiculum volume	0.010	*t*(29) = −2.758
Pu et al. [14]	55.46 ± 7.37	1000 m	≈4 years	Green space (correlated with blue space)	Green space = (45.21% ± 21.58% at 1000 m buffer) contributed to 39.4% of the variance in brain volume, 49% in white matter volume, and 21.7% in gray matter volume.	Precuneus	0.002	β = 0.006
					Blue space = (1.26% ± 2.46% at 1000 m buffer) contributed to 6.3% of the variance in brain volume, 12.1% in white matter volume, and 3.8% in gray matter volume.	—	Not sig.	—
Shang et al. [15]	54.7 ± 7.5 at baseline; 63.5 ± 7.6 years at MRI	300 m and 1000 m	8.8 years	Residential greenspace	Each 10% increase in greenness at 1000 m.	a. Total brain b. Grey matter c. White matter	a. 0.0065 b. 0.0004 c. 0.0014	a. β = 0.013 (95% CI: 0.005, 0.020) b. β = 0.013 (95% CI: 0.006, 0.020) c. β = 0.011 (95% CI: 0.004, 0.017)
				Natural environment	Each 10% increase in natural environment at 1000 m.	a. Total brain b. Grey matter c. White matter	a. Not sig. b. 0.0084 c. 0.0040	a. β = 0.010 (95% CI: 0.004, 0.017) b. β = 0.009 (95% CI: 0.004, 0.015) c. β = 0.010 (95% CI: 0.004, 0.016)
Baena-Extremera et al. [16]	Green exercise: 39.91 ± 6.35; Urban exercise: 40.00 ± 6.19	N/A	>5 years	Natural environment	Exercise in mountains, valleys, and forests.	a. rACC b. rSFS	a. 0.034 b. 0.037	a. t = 2.181 b. t = 2.151
Binter et al. [17]	Gen. R cohort: 10.1 ± 0.6; PELAGIE cohort: 10.8 ± 0.3	300 m; distance to green space and blue space and area	9 years	NDVI, Distance to public green space, and public green space area	Greater distance to the nearest major green space during pregnancy was associated with higher whole-brain FA per 7 m increase.	Whole-brain FA	—	a. β = 0.001 (95%CI 0.000; 0.002)
Webb et al. [18]	36.5 ± 13.4	100 m	2 weeks	NDVI	NDVI was associated with greater neural reactivity to reward in the amygdala.	Amygdala	0.02	β = 0.18; *t*_277_ = 2.83
Besser et al. [19]	75 ± 4.4	1000 m	≈ 5 years	NLCD	Greenspace alone (split at a median of 37%) was insignificant.	a. WM grade b. Ventricle grade	Not sig: a. 0.09 b. 0.49	a. OR = 1.36 (95% CI: 0.95–1.95) b. OR = 0.88 (95% CI: 0.62–1.26)
Kühn et al. [20]	12.78 ± 0.27	200 m, 500 m, and 1000 m	12.5 years	TCD, CLC (open green space with the sky view factor (SVF))	TCD: 9.41% ± 5.43% (200 m buffer) negatively associated with many GM clusters that were visible at the 500 m radius but smaller. No significance at the 1000 m radius.	Clusters within the fronto-median wall, bilateral lateral prefrontal cortex, right middle temporal and left middle occipital gyrus.	<0.001	Negative associations with Medial orbitofrontal cortex, left and medial superior frontal gyrus, right middle frontal gyrus, right middle temporal gyrus, anterior cingulate cortex, right inferior frontal gyrus, left middle occipital gyrus, right inferior frontal gyrus, left middle frontal gyrus.
					OGS: 8.73% ± 14.32% (200 m buffer) had positive associations with many GM clusters. Clusters were visible at the 500 m radius but smaller, but there was no significance at the 1000 m radius.	OSG: Clusters within the bilateral prefrontal cortex and temporal cortex.	<0.001	Positive associations with Left middle temporal gyrus/occipital middle gyrus, right dorsolateral prefrontal cortex, right superior temporal gyrus, left and right inferior and right middle frontal gyrus, left middle temporal gyrus, left middle occipital gyrus, left middle occipital gyrus, left middle frontal gyrus.
						SVF: Blitaletral areas and parietal lobe.	<0.001	Positive associations with Left inferior parietal lobe, right parahippocampal gyrus, left frontal mid orbital gyrus, left inferior parietal lobe, left superior frontal gyrus, right middle temporal gyrus, right superior frontal gyrus, right middle frontal gyrus, left frontal mid orbital gyrus, left inferior parietal lobe, left superior medial frontal, left inferior parietal lobe, left superior medial frontal.
Kühn et al. [21]	22.1 ± 0.67	500 m, 1000 m, and 2000 m	—	TCD	TCD = 10.12% (500 m). No significant findings for 1000 m (10.79%) and 2000 m (12.02%).	Right orbitofrontal cortex (rOFC) GM.	<0.001	—
Gianaros et al. [22]	42.52 ± 0.35	—	—	Green space percentage <40% and ≥40%	≥40% greenness.	a. Cortical volume b. Hippocampal volume	Indirect	Mediated by cardiometabolic risk, and weaker for hippocampal volume than cortical volume.
Tani et al. [23]	70.1 ± 5.4	1000 m	30 years	Subjective and objective (NDVI, green space, blue space)	Subjective beauty, which was higher for a mountain area than a downtown area.	a. mOFC b. Insula	a. mOFC: 0.001 left, 0.01 right b. Insula: 0.002 left, <0.0001 right.	a. 242 (95% CI 118, 366) in the left mOFC, and 166 (95% CI 47, 286) in the right mOFC b. 218 (95% CI 79, 356) in the left insula, and 317 (95% CI 162, 472) in the right insula.
Sudimac et al. [24]	27.21 ± 6.61	—	1 h	Forest	Walking in the forest, not the urban environment.	Amygdala	0.014	*t*(31) = 2.62
Sudimac and Kühn [25]	27.21 ± 6.61	—	1 h	Forest	Women walking in the forest, not men.	Amygdala	0.034	*t*(14) = 2.35
Kühn et al. [26]	70.1 ± 3.77	1000 m	—	Urban green	Urban green 6.79% ± 9.03%.	Perigenual/subgenual anterior cingulate cortex (p/sACC)	<0.001	13% of the variance in the volume of p/sACC is explained by adding urban green to the regression model.
Besser et al. [27]	79.1 ± 4.1	1000 m and 5000 m	5 years	NLCD for 1000 m buffer	Greenspace: 38.3% (SD = 27.6%) 5 y. before MRI, 29.5% (SD = 26.6%) at MRI.	a. Hippocampus b. ventricle grade	a. Not sig. b. 0.06	a. — b. −0.30 (95% CI: −0.61, 0.00)
Min et al. [28]	68.0 ± 10.1	750 m	1 year	EVI	Urban EVI = 0.28	a. Global cortical thickness b. Parietal cortex c. Occipital cortex d. Frontal cortex	a. 0.038 b. 0.009 c. 0.021 d. Not sig.	a. 7 μm (CI: 0–13 μm) b. 11 μm (3–20 μm) c. 9 μm (1–16 μm) —
Falcón et al. [29]	58.6 ± 7.5	300 m	>3 years	NDVI	—	a. Middle frontal cortex b. Left precentral c. Right temporal	<0.001 (uncorrected)	a. 441 voxels b. 528 voxels c. 730 voxels
Crous-Bou et al. [30]	57.7 ± 7.6	300 m	>3 years	NDVI	NDVI: 0.22 ± 0.06	a. Cortical thickness b. Hippocampal volume c. ventricles volume	a. 0.03 b. Not sig. c. Not sig.	a. β = 0.08
Dzhambov et al. [31]	49.73 ± 3.20	500 m	>5 years	NDVI	NDVI: 0.40 ± 0.08	Cortical thickness	Not sig.	—
Dadvand et al. [32]	8.4 ± 1.3	100 m	≈ 8.5	NDVI	NDVI: 0.10 ± 0.06	Prefrontal cortex, premotor cortex, cerebellum	all < 0.05	GM volume: Left prefrontal cortex (*t* = 3.05) Right prefrontal cortex (*t* = 2.7) Left premotor cortex (*t* = 3.05) WM volume: Right prefrontal region (*t* = 3.04) Left premotor region (*t* = 2.97) Cerebellum: left (*t* = 3.34) Cerebellum: right (*t* = 3.26).

* No studies available.

## Data Availability

All data extracted are presented in the current document. No other external materials are available.

## Abbreviations

FA	Fractional Anisotropy
GM	Grey Matter
WM	White Matter
TCD	Tree Cover Density
SVF	Sky View Factor
NDVI	Normalised Difference Vegetation Index
EVI	Enhanced Vegetation Index
NLCD	National Land Cover Database
CLC	Corine Land Cover
AD	Alzheimer’s Disease
ACC	Anterior Cingulate Cortex
rACC	Right Anterior Cingulate Cortex
rSFS	Right Superior Frontal Sulcus
OFC	Orbitofrontal Cortex
mOFC	Medial Orbitofrontal Cortex
rOFC	Right Orbitofrontal Cortex
p/sACC	Perigenual/Subgenual Anterior Cingulate Cortex
